# Dietary and Other Risk Factors in The Aetiology of Cholelithiasis: A Case Control Study

**DOI:** 10.1155/1989/56539

**Published:** 1989

**Authors:** A. D. Linos, V. Daras, D. A. Linos, V. Kekis, M. M. Tsoukas, V. Golematis

**Affiliations:** 1 Department of Epidemiology Athens Medical School NIKE Hospital Greece; 2 Department of Surgery Athens Medical School NIKE Hospital Greece; 3 Athens Medical School NIKE Hospital Greece; 4 University of Athens 110 Vasilissis Sofias Avenue Athens GR-11527 Greece

## Abstract

We studied the effect of dietary factors and a variety of other risk factors on the development of cholelithiasis through a case control study.

The study involved 96 cases and 118 age and sex matched controls. All cases and controls were interviewed with regard to a variety of risk factors and frequency of consumption of over 100 food items. Analysis was done both by chi square and a multiple logistic regression model. From all the dietry factors the only ones that showed a positive statistically signficantly (p<0.05) association was consumption of animal fat as expressed by eating all visible fat on the meat and using butter on the table. Interestingly high consumption of olive oil had a negative (protective) association with the disease. A negative association was also found with smoking and holding a job demanding hard labor.


*HPB Surgery* 1989, Vol. 1, pp. 221–227
Reprints available directly from the publisher
Photocopying permitted by license only

© 1989 Harwood Academic Publishers GmbH
Printed in Great Britain

### DIETARY AND OTHER RISK FACTORS IN THE AETIOLOGY OF CHOLELITHIASIS: A CASE CONTROL STUDY

A.D. LINOS*, V. DARAS***, D.A. LINOS**, V. KEKIS***, M.M. TSOUKAS** and V.GOLEMATIS**

*Departments of Epidemiology* and Surgery** Athens Medical School NIKE Hospital****

*(Received 23 December 1988)*

We studied the effect of dietary factors and a variety of other risk factors on the development of cholelithiasis through a case control study.

The study involved 96 cases and 118 age and sex matched controls. All cases and controls were interviewed with regard to a variety of risk factors and frequency of consumption of over 100 food items. Analysis was done both by chi square and a multiple logistic regression model. From all the dietry factors the only ones that showed a positive statistically signficantly ($p<0.05$) association was consumption of animal fat as expressed by eating all visible fat on the meat and using butter on the table. Interestingly high consumption of olive oil had a negative (protective) association with the disease. A negative association was also found with smoking and holding a job demanding hard labor.

KEY WORDS: Cholelithiasis, Diet, Olive oil

### INTRODUCTION

Traditionally it has been thought that cholelithiasis is the disease of the five Fs: fat, forty fair, female, fertile. Yet few studies [1-5] have addressed the issue of obesity, or have investigated the possibility that some underlying factor such as specific dietary habits are implicated in the development of the disease. Furthermore, evidence from these studies is conflicting. Thus Smith and Gee [1] found that cholelithiasis cases were overweight but consumed less protein, fat and carbohydrate whereas Scragg et al [2] reported that obesity was associated with the disease only in young persons who consumed more fat. Furthermore they observed a decreased risk of the disease with increased use of alcohol although no effort was made to assess smoking, a factor highly correlated with alcohol consumption.

In order to investigate the role of diet, smoking and other factors on the development of cholelithiasis we performed a case control study of cholelithiasis and dietary habits in Greece, a country with great diversity of diets ranging from a rather traditional dietary pattern where vegetables and olive oil are predominant to complementary westernized diets where animal protein and fat are the major food items.

* Correspondence to: Dr. Athena Linos, University of Athens, 110 Vasilissis Sofias Avenue, GR-11527, Athens, Greece.

### MATERIALS AND METHODS

We studied 96 cases of newly diagnosed cholelithiasis and 118 controls, serially matched to the cases by age and sex.

Cases were drawn from two major hospitals in the Athens metropolitan area (one public hospital serving low and middle class patients and a private hospital serving upper middle class and high socio-economic class patients). Both hospitals provide care to patients from the Athens metropolitan area as well as from many other areas in Greece.

Diagnosis of cholelithiasis in all cases was verified by radiologic examinations and subsequent surgery. Controls were drawn from the same hospitals as the cases, and were group matched to the cases by age, sex and hospital, by group matched we mean that controls were chosen so that their distribution by age and sex were similar to the cases.

The majority of the controls were also surgical patients. Any patient with a disease known or suspected to be related to diet (e.g. colon cancer, other large bowel disease etc.) was excluded from the control pool. All other patients were included so that the possibility for introducing bias via the way controls were selected was minimized.

All cases and controls were interviewed while still in the hospital. The interview included detailed information regarding basic demographic data (age, sex, place of birth, place of permanent residence, marital status), socioeconomic data (occupation, years of schooling completed), basic vital data (height, weight, birth order), medical data (previous hospitalizations, surgery and a number of relevant diagnoses), data on social habits (smoking, drinking, coffee and tea consumption) and finally information on frequency of use (ranging from once or twice a month to daily use) of over 100 food items. Futhermore we asked specific questions regarding additional use of vegetable and animal fat. All questions referred to habits prior to the diagnosis cholelithiasis or date of control selection. For analysis food items were grouped into ten major groups (meats and sea foods, milk and cheese, bread and cereals, vegetables, fruits, pulses, fats and oils, pastries and finally non-alcoholic beverages). On the basis of height and weight we calculated Quetelet's body mass index (weight/height$^2$). The present paper presents analysis regarding the data referring to dietary and social habits as well as basic demographic variables that could act as confounding factors.

We performed both univariate and multiple logistic analysis. For univariate analysis all tests were done with the chi square statistic. For multivariate analysis we used the model developed by Breslow and Day [6]. During the multiple logistic analysis development of the disease was considered the dependent variable and as independent variables we used age, sex, body mass index, place of permanent address, place of birth, occupation, smoking (measured as pack(s) × years accumulated up to date of diagnosis) years of school attended, coffee, tea and alcohol consumption and the ten food groups presented previously.

This way all independent variables could not act as confounding factors when the relationship of each one of them with the disease was examined.

### RESULTS

Of the 96 cases of cholelithiasis studied 43 were men and 53 women. Of the 118

controls 60 were men and 58 were women. Although the matching by sex is not perfect the differences were not statistically significant ($X^2 = 0.77$ $p > 0.20$). There were no differences in the age distribution of the cases and the controls. With regards to the place of birth and place of residence there were some noticeable differences in that cases were more often residents of Athens than controls and the same holds true for place of birth. The differences though were of boderline statistical significance ($p<0.10$). Since these differences could affect the dietary habits of cases and control place of birth and place of residence were included in the multiple logistic model as independent variables. Similarly there were differences of borderline statistical significance in the marital status of case and controls. More specifically 92% of the cases were married whereas 81% of the controls were married. This finding along with other reproductive findings will be presented elsewhere.

With regards to occupation people were grouped in seven basic categories a) farmers and gardeners b) labourers c) white-collar workers d) professionals e) merchants and store owners f) housewifes, retirees and g) other. The distribution of cases and controls according to this categorization of occupations is presented on table 1. It is obvious from this table that the controls more often held jobs that required more manual work "farmer", "labourer", "white-collar worker" than the cases. The difference was statistically significant ($X^2 = 23.6$ $p<0,001$). Given that these occupations also belong to lower socioeconomic classes the differences in the residency of cases and controls and the fact that group 6 (where some over representation of the cases is evident) includes housewives, it is hard to decide whether the differences indicate some direct association with the disease or other socioeconomic differences.

**Table 1** Distribution of cases and controls according to occupation.

| | *Farm.* | *Labor.* | *Whtc. Work.* | *Prof/ nals* | *Merch. St. Ow.* | *Hswives & Ret.* | *Other* | *Total* |
|---|---|---|---|---|---|---|---|---|
| Cases | 8 | 9 | 12 | 6 | 11 | 47 | 2 | 95 |
| Controls | 25 | 19 | 30 | 5 | 4 | 34 | 1 | 118 |
| Total | 33 | 28 | 42 | 11 | 15 | 81 | 3 | 213 |

Table 2 presents distribution of cases and controls according to smoking habits; again the differences are substantial mainly in terms of over representation of the controls in the highest smoking category. The overall $X^2$ was 10.31 ($p<0.02$).

Table 3 presents the distribution of cases and controls according to body mass index as measured by the Quetelet's body mass index. For table presentation, cases and controls were grouped in 4 groups. Group 1 being the persons with the lowest indexes and group 4 persons with the highest indexes. As can be seen in the table there are some differences, mainly in the two extreme groups but these differences do not approach statistical significance. Furthermore to interpret these data controlling by age and sex is necessary. Therefore we consider it more appropriate to draw our conclusion from the multiple logistic analysis where exact values of the index are used.

Table 4 presents similar data with regards to alcohol consumption. Again there is over representation of the control in the two highest categories. The overall $X^2$ is of borderline statistical significance.

**Table 2** Distribution of cases and controls according to their smoking habits.

| *Smoking category* | *Never smoked* | *1–10 Packs X year* | *11–30 Packs X year* | *31+ Packs X year* | *Total* |
|---|---|---|---|---|---|
| Cases | 53 | 15 | 15 | 13 | 96 |
| Controls | 51 | 16 | 13 | 38 | 118 |
| Total | 104 | 31 | 28 | 51 | 214 |

**Table 3** Distribution of cases and controls according to body mass index.

| *BMI (Kg/m²)* | *– 16.5* | *16.5–23.0* | *23.0–25.3* | *25.3–27.8* | *27.8+* | |
|---|---|---|---|---|---|---|
| Cases | 1 | 22 | 21 | 21 | 28 | 93 |
| Controls | 4 | 39 | 27 | 25 | 23 | 118 |
| Total | 5 | 61 | 48 | 46 | 51 | 211 |

**Table 4** Distribution of cases and controls according to their alcohol consumption.

| *Alcohol consmp[a].* | *None* | *1–9* | *10–19* | *20+* | |
|---|---|---|---|---|---|
| Cases | 43 | 36 | 8 | 7 | 94 |
| Controls | 51 | 32 | 11 | 23 | 117 |
| Total | 94 | 68 | 19 | 20 | 211 |

[a]Measured in glasses per week

As far as specific dietary groups are concerned from the ten groups examined in the univariate analysis we did not find differences between cases and controls in the consumption of meats and sea foods ($p>0,20$), milk products ($p>0.50$), breads and cereals ($p>0.90$), vegetables ($p>0.50$), pulses ($p>0.10$) and beverages ($p>0.10$). On the contrary we found significant differences in the use of fruits [Cases ate more fruit than controls ($p<0.05$)] and in the use of pastries and cakes [(cases also ate more pastries and cakes ($p<0.05$)].

As far as fat consumption is concerned cases consumed significantly more fat of animal origin. Thus 24% of the cases and 10% of the controls (table 5) ate all visible fat on meat, whereas 10% of cases and 3% of controls used additional butter on the table. On the contrary olive oil was added to food more frequently by controls than by cases. More specifically 48% of controls and 43% of cases used added olive oil in their food. Although one could argue that all above findings are valid they could also easily be the result of several possible confounding factors such as overall caloric consumption, socioeconomic differences and consumption of other food items. To control for this possibility we performed multiple logistic analysis.

Table 6 presents the factors which showed a significant association with the disease in the logistic analysis. One should mention here that the statistically significant associations observed in logistic analysis remain significant even after controlling for the possible confounding action of all the factors that were included in the model (mentioned in materials and methods.)

**Table 5** Distribution of cases and controls according to their comsumption of visible fat on meat.

| *Fats consumption* | *Eats no vis. fat* | *Eats some vis. fat* | *Eats all vis. fat* | *Total* |
|---|---|---|---|---|
| Cases | 70 | 3 | 23 | 96 |
| Control | 104 | 2 | 12 | 118 |
| Total | 174 | 5 | 35 | 214 |

**Table 6** Results of multiple logistic analysis in the study of dietary risk factors and cholelithiasis.

| *Risk factor* | *p* | *RR*[a] | *(95% CI)*[b] |
|---|---|---|---|
| *Butter consumption* | *p*<0.05 | 10.9 | (1.3–92.5) |
| Animal fat cosumption | p<0.01 | 1.9 | (1.04–3.5) |
| Olive oil consumption | p<0.10 | 0.4 | (1.15–1.09) |
| Smoking (10 pack × years diff) | p<0.01 | 0.7 | (0.70–0.73) |
| Manual work occupation | p<0.05 | 0.2 | (0.05–0.45) |

[a] Relative Risk of Developing the disease if factor exists as compared to when it does not exist.
[b] 95% confidence intervals of the Relative Risk.

Thus factors for which no association with the disease was found in logistic analysis included body mass index, place of residence and place of birth, years of school attended, alcohol, coffee and tea consumption and all the food groups. Age and sex were also included in the model so that we could control for their potential confounding action. Their relationship to the disease could not be examined in this study because we matched by these factors and because the cases do not come from a closed population and therefore they are not incidence data.

As can be seen from table 6 the only factors that showed a statistically significant positive association with the disease is the consumption of animal fat as expressed by the use of butter and eating all visible fat on meat. Interestingly enough a statistically significant signficant negative association was observed with smoking ($p<0.01$), and holding a job of a blue or white collars worker ($p<0.05$). Whereas a negative association of border line statistical significance was found with olive oil consumption ($p<0.10$).

Given that there were no differences in the body mass index found (when controlling for age, sex and types of food consumed) one can conclude that obesity *per se* has no effect on the disease development, but animal fat has a positive relationship with the disease.

### DISCUSSION

The present study is the first to our knowledge in which the issue of the effect of types of fat consumed (especially olive oil) and smoking on the development of cholelithiasis was addressed while the majority of other known risk factors were controlled either through the study design or in the analysis. It is of interest that the only factor that showed a positive statistically significant association with the disease was the consumption of animal fat either as fat contained in meat or as butter added in food or eaten on the table. Interestingly enough vegetable oils other than olive oil have been only recently introduced in the Greek diet and therefore the contrast between olive oil and animal fat was more easily made. Our finding agrees in part with the finding of Scragg et al [2] according to which more fat was consumed by young cholelithiasis patients than by their controls. In our study 30% of the subjects were under age 50, but the finding of increased risk only with animal fats may in part explain the conflict in the two previous studies [1,2] if one considers that they came from two different continents where types of fats used may differ. It is of great interest that a protective effect was found with the use of olive oil. Olive oil contains mainly the monounsaturated long carbon chain length (C-18) oleic acid, a modest percentage of the polyunsaturated linoleic and linolenic fatty acids and a small percentage of saturated fatty acid (palmitic and stearic). Olive oil also contains phytosterols especially --sitosterol [7].

Olive oil especially through the action of oleic acid stimulates the secretion of cholecystokinin which increases the tone of the gallbladder, and relaxes the sphincter of Oddi [7,8]. Malagelada et al studying the effects of fatty acid chain length on pancreatic and gallbladder function in healthy volunteers found that pancreatic and gallbladder responses were augmented by increasing fatty acid chain length [9]. Thus, a beneficial effect of the long chain length oleic acid on the emptying of gallbladder can be postulated. On the other hand, --sitosterol which is especially high in concentration in olive oil is known to reduce plasma cholesterol by interfering with intestinal absorption of cholesterol [10]. In fact --sitosterol administered to human volunteers resulted in 12% mean decrease in biliary cholesterol saturation after 6–8 weeks [11]. Thus olive oil may play a protective role in the pathogenesis of cholelithiasis either through its cholagogic action or by reducing the biliary cholesterol concentration via the --sitosterol action. Interestingly enough data from Italy suggest that cholelithiasis is less frequent in the areas in Italy where olive oil consumption was higher [7].

Despite the common impression, we found that obesity, as expressed by body mass index, was not associated with disease although animal fat consumption was.

It is of interest that a major study done in Rochester Minn [5]where incidence cases were used, showed also, no association between obesity and gall bladder disease.

In our study no relationship with alcohol consumption was found whereas in the study of Scragg et al [2] a decrease of the risk of cholelithiasis with alcohol consumption was reported. We found, however a substantial decrease in the risk of cholelithiasis with smoking. A possible explanation for the apparent discrepancy between these studies is that alcohol consumption and smoking are interrelated (people who smoke usually drink too). Therefore smoking could have acted as a confounding factor in the study of Scragg et al who did not control for smoking in their multivariate analysis.

Finally in our study a strong negative relationship was also found with occupations involving hard manual work. Given though the fact that these same occupations are associated with lower socioeconomic class the finding should be interpreted cautiously, since some inter relationships with diet may also exist.

### *References*

1. Smith, D.A. and Gee, M.J. (1979) A dietary survey to determine the relationship between diet and cholelithiasis. *Am. J. Clinic Nutr.*, **32**, 1519–26

2. Scragg, R.K.R., Mc Michael, A.S. and Baghurst, P.A. (1984) Diet Alcohol and relative weight in Gallstone Disease. A case control study, *Br. Med. J.* **288**, 1113–19
3. Van Der Linden, W. (1961) Some biological traits in Female gall stone Disease patients. *Acta Chir Scand suppl.* **269**, 1–94
4. Sampliner, R.E., Bennett, P.H., Comess, L.J., Rose, F.A. and Burch, T.A. (1970) Gall bladder disease in Pimo Indians. Demonstrated of High prevalence and early onset by cholecystography. *New Eng. J. Med.*, **283**, 1398–67
5. Maram, S.E., Thistle, J.L., Linos, A.D., Linos, D.A., O'Fallon, W.M. and Kurland, L.T. (1988) Pregnancy and obesity as risk factors for the development of cholelithiasis. (Submitted)
6. Breslow, N.E. and Day, N.E. Statistical methods in cancer research Vol. 1 The analysis of case-control studies IARC Scientific Publications No 32, IARC Lyon 1980
7. Viola, P. and Audisio, M. (1987) Olive oil and Health International olive oil Council Madrid Spain, pp. 18–19
8. Shiratori, K., Watanabe, S., Chey, W.Y., Lee, K.Y. and T.M. Chang (1973) Endogenous cholecystokinin drives gallbladder emptying in dogs. *Surgery,* **73**(266), 553–58
9. Malagelda, J.R., DiMagno, E.P., Summerskill, W.H.J. and Go V.L.W. (1976) Intraluminal fatty acids and bile acids in man *J. Clin Invest* **58**, 493–99
10. Grundy, S.M. and Mok, HYI (1976) In Greten H (Ed), Lipoprotein Metabolism Springer, Heidelberg, pp. 112–18
11. Begemann, F., Bandomer, G. and Herget, H.J. (1978) The influence of --sitosterol on biliary cholesterol saturation and bile acid kinetics in man. *Scand. J. Gastroent.* **13**, 57–63

*Accepted by S. Bengmark 18 July 1988.*

